# Preparation and Reinforcement of Dual‐Porous Biocompatible Cellulose Scaffolds for Tissue Engineering

**DOI:** 10.1002/mame.201500048

**Published:** 2015-04-28

**Authors:** Nicole Pircher, David Fischhuber, Leticia Carbajal, Christine Strauß, Jean‐Marie Nedelec, Cornelia Kasper, Thomas Rosenau, Falk Liebner

**Affiliations:** ^1^ Department of ChemistryDivision of Chemistry of RenewablesUniversity of Natural Resources and Life Sciences ViennaKonrad‐Lorenz‐Straße 24, 3430 TullnViennaAustria; ^2^ Institute of Chemistry of Clermont‐FerrandClermont UniversitéEcole Nationale Supérieure de Chimie de Clermont‐FerrandBP 1044863000Clermont‐FerrandFrance; ^3^ Institute of Chemistry of Clermont‐FerrandCentre National de la Recherche Scientifique24 av. des Landais63171AubièreFrance; ^4^ Department for BiotechnologyUniversity of Natural Resources and Life Sciences ViennaMuthgasse 18, 1190 WienViennaAustria

**Keywords:** cellulose aerogels, dual‐porosity, porogens, supercritical anti‐solvent precipitation, tissue engineering

## Abstract

Biocompatible cellulose‐based aerogels composed of nanoporous struts, which embed interconnected voids of controlled micron‐size, have been prepared employing temporary templates of fused porogens, reinforcement by interpenetrating PMMA networks and supercritical carbon dioxide drying. Different combinations of cellulose solvent (Ca(SCN)_2_/H_2_O/LiCl or [EMIm][OAc]/DMSO) and anti‐solvent (EtOH), porogen type (paraffin wax or PMMA spheres) and porogen size (various fractions in the range of 100–500 μm) as well as intensity of PMMA reinforcement have been investigated to tailor the materials for cell scaffolding applications. All aerogels exhibited an open and dual porosity (micronporosity >100 μm and nanoporosity extending to the low micrometer range). Mechanical properties of the dual‐porous aerogels under compressive stress were considerably improved by introduction of interpenetrating PMMA networks. The effect of the reinforcing polymer on attachment, spreading, and proliferation of NIH 3T3 fibroblast cells, cultivated on selected dual‐porous aerogels to pre‐evaluate their biocompatibility was similarly positive.
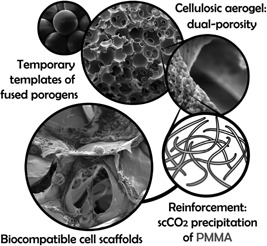

## 1 Introducion

Immune rejection and disease transmittal are just two examples of significant risks still related to autologous or allogeneic tissue grafts.^1^ In tissue engineering, synthetic extracellular matrices (ECM) are used as scaffolds to regenerate a patient's own tissue, an approach highly beneficial in terms of biocompatibility and biofunctionality. Driven by new insights into the complex requirements of ECM scaffolds for tissue engineering and novel synthetic and analytical approaches, a multitude of inorganic and organic materials including composites has been developed and investigated.^1^, ^2^

Development of biocompatible synthetic ECMs is a challenging endeavor as respective materials have to meet a series of morphological, biomedical, and mechanical requirements. Adequate interconnected porosity and nanostructured surfaces are essential prerequisites to facilitate cell migration, attachment, and in‐growth in a three‐dimensional (3D) scaffold, neovascularization as well as diffusion of physiological nutrients and gases to cells and removal of metabolic by‐products. Due to cell size, migration requirements, and capillary formation, interconnected, large pores of at least 100 μm, better >200 μm in diameter (“micronporosity”) are essential for cell in‐growth and proliferation.^1^, ^2^, ^3^ The simultaneous presence of smaller pores in the low micrometer range and the full nanometer scale (“nanoporosity”) can greatly enhance the scaffolding performance and is essential for diffusion of physiological nutrients and gases to the cells or the removal of metabolic by‐products. In the case of bone tissue engineering, their presence alongside large micron‐size pores has been demonstrated to induce lamellar and woven bone formation in hydroxyapatite scaffolds.^1^, ^2^^]^^[1a,2]^ Aside from cell adhesion, the presence of interconnected smaller pores is beneficial for *in vivo* neovascularization by allowing capillary in‐growth throughout the scaffold.^3^ Nanoporosity is also accompanied by a larger surface area which could contribute to higher protein adsorption, ion exchange, and hydroxyapatite formation in tissue.^1^, ^4^

Surface morphology of scaffolds is another factor that largely impacts cellular response. In contrast to flat and rigid surfaces, a 3D, nanofibrous topography significantly promotes interactions between cells and the extracellular matrix.^3^ Previous studies have shown that coagulation of cellulose from low‐concentration solution state yields highly porous, polymorphic cellulose II suprastructures (networks or agglomerated spheres of entangled nanofibrils, depending on the type of solvent used). The open pore system often constitutes more than 95 vol%, and pore diameters are broadly distributed across the entire nanoscale up to the low micron range. Considering the aforementioned benefits of nanoporosity and nanostructured surfaces, the particular morphology of cellulose hydro‐ and aerogels renders them promising cell scaffolding materials. Cellulose and a multiplicity of cellulose derivatives show good biocompatibility and can be, as observed for some derivatives, bioresorbable.^5^

Since the preparation of cellulose lyogels is accomplished by antisolvent‐mediated coagulation of the polysaccharide from solution state, respective micronporous gels can supposedly be obtained by incorporation of porogens of tailored size and shape.^6^ However, increased micron‐scale is typically associated with a loss of mechanical robustness, such as compressive strength and Young's modulus.^2^, ^7^ Therefore, reinforcement strategies are additionally required to strengthen the cellulosic network.

In the present work, fused paraffin wax and poly(methyl methacrylate) (PMMA) spheres have been used as temporary templates to generate an interconnected, dual porosity during coagulation of cellulose. While the morphology and nanopore characteristics of the cellulose II network forming the scaffold struts was controlled by the choice of cellulose solvent, the pore size distribution of large micronpores (up to several 100 µm), was set within the range of 100–500 μm through the choice of porogen particle size fraction (100–200, 200–300, 300–500 μm).

After leaching of the temporary scaffold of fused porogens, the fragile, dual‐porous scaffolds were reinforced by an interpenetrating PMMA network, consecutively using supercritical carbon dioxide (scCO_2_) anti‐solvent precipitation and drying techniques. Due to its biocompatibility and good mechanical properties, PMMA has been well established in biomedical applications, especially for treatment of bone defects, for example, as a component of bone cements.^8^ In contrast to cellulosic materials, whose rate of bioresorbability depends on the degree of crystallinity and can be adjusted through type and degree of derivatization (e.g., oxidation, hydroxyethylation),^5^, ^9^ PMMA is biostable. Accordingly, microfibrillar cellulose II networks constitute cell scaffolding materials of customizable pore structure, high surface area, and nanostructured surface features, and the addition of biocompatible PMMA as a secondary, persistent constituent guarantees long‐term mechanical stability after implantation.^10^

## 2 Experimental Section

### 2.1 Materials

Ammonium thiocyanate, lithium chloride (LiCl), poly(methyl methacrylate) (PMMA, *M*_w_ ∼350.0 kg mol^−1^), paraffin wax (mp 53–57 °C), poly (vinyl alcohol) (PVA, *M*_w_ 31–35 kg mol^−1^, 98–99% hydrolyzed), 3‐(4,5‐dimethylthiazol‐2‐yl)‐2,5‐diphenyltetrazolium bromide (MTT), 4′,6‐diamidino‐2‐phenylindole (DAPI), and Dulbecco's Modified Eagle's Medium (DMEM) were purchased from Sigma–Aldrich. Calcium hydroxide was obtained from Fluka Analytical. Dimethylsulfoxide (DMSO) and hydrochloric acid (HCl) were purchased from Merck. 1‐Ethyl‐3‐methylimidazolium acetate ([EMIm][OAc]) was a donation of BASF (Ludwigshafen, Germany). Absolute ethanol was purchased from Fisher Scientific. Analytical grade acetone and tetrahydrofuran (THF) were obtained from VWR. Natal calf serum (NCS) and penicillin‐streptomycin (P/S) were purchased from PAA Laboratories, phosphate buffered saline (PBS) from Life Technologies, and sodium dodecyl sulfate (SDS) from Applichem. NIH 3T3 fibroblast cells were obtained from The Leibniz Institute DSMZ—German Collection of Microorganisms and Cell Cultures.

### 2.2 Temporary Scaffolds of Fused Porogen Spheres

#### 2.2.1 Paraffin

Paraffin microspheres were obtained by solidification of wax droplets, emulsified in an aqueous solution of PVA.^11^ Briefly, 5 w/vol% PVA were suspended in 400 ml deionized water and heated to 90 °C. After 2 h, a clear aqueous PVA solution was obtained. 20 g paraffin wax, molten at 90 °C, was added and the mixture was vigorously stirred to form an emulsion. To solidify the wax droplets, 1.5 l of ice‐cold‐deionized water was added under thorough stirring. The mixture of paraffin wax spheres, dispersed in water was then poured through a sieve (mesh size: 100 μm) and rinsed with deionized water to remove PVA. Afterwards, the microspheres were fractionized under deionized water flow according to their size using sieves with mesh sizes of 100, 200, 300, and 500 μm. The individual fractions (100–200, 200–300, and 300–500 μm) were frozen at –80 °C and lyophilized. For the preparation of the temporary template, the respective size fraction of porogen spheres was evenly filled into 5 or 10 ml glass syringes equipped with a removable needle adapter. Interconnections between the microspheres were generated through compaction of the loosely packed porogen bed by about 17 vol% using a syringe piston. The contact points of the spheres were then fused at 44 °C for 40 min.

#### 2.2.2 PMMA

Scanning electron microscopy (SEM) of the purchased poly(methyl methacrylate) powder revealed microspheres with diameters largely ranging from 100 to 300 μm. Therefore, PMMA was directly wet‐screened under deionized water to obtain respective size fractions of 100–200 μm and 200–300 μm. After deep‐freezing (−80 °C) the samples were lyophilized.

**Figure 1:**
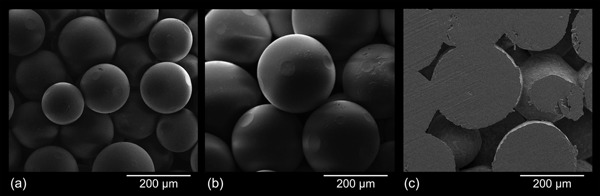
SEM micrographs of fused porogen spheres prior to filling the temporary scaffolds with respective cellulose solutions: PMMA size fractions 100–200 μm (a), 200–300 μm (b), and paraffin wax 200–300 μm (c).

As for the paraffin wax porogens, the fractionized and dried PMMA powder was filled into glass syringes, heated to 150 °C for 40 min, and compacted by about 17 vol% using a syringe piston. Fusion of the PMMA spheres was accomplished at 150 °C (20 min). In comparison to the obtained paraffin scaffolds, the contact points between the PMMA spheres were relatively small (Figure 1b and c). This drawback, however, was compensated somewhat later in the preparation procedure when PMMA was leached from the samples as swelling of the spheres prior to their dissolution caused a considerable increase of the micronpore‐interconnections.

### 2.3 Dissolution of Cellulose

Calcium thiocyanate was prepared from Ca(OH)_2_ (calcium hydroxide) and NH_4_SCN (ammonium thiocyanate). One mole Ca(OH)_2_ was slurried in 300 ml deionized water and mixed with the same amount of an aqueous solution of 2 mol NH_4_SCN. The mixture was concentrated to about 250 ml by slow evaporation of aqueous ammonia and another 500 ml of deionized water were added. This step was repeated until the solution became neutral. After filtration to remove precipitated calcium carbonate and impurities, the clear aqueous solution of Ca(SCN)_2_ was concentrated to about 200 ml under vacuum (45 °C, 9 mbar). The product was crystallized by cooling to −20 °C and subsequently freeze–dried. To determine the water content of the final product, a small amount was dissolved in absolute ethanol and analyzed by Karl–Fischer titration.

Regenerated cellulose gels were prepared from respective solutions in Ca(SCN)_2_/H_2_O/LiCl (C/P/x) or [EMIm][OAc]/DMSO (E/x/x) using cotton linters (CL, *M*_w_ 143.2 kg mol^−1^) as raw material.

C/P/x series: 1.5 wt% CL was dissolved at 140 °C in a mixture of calcium thiocyanate, deionized water, and 2.5 wt% LiCl. The water content of this particular cellulose solvent was adjusted to 8 mol water per mole calcium thiocyanate to account for the highest coordination number of the calcium cation. Such ratios lead to preferred ionic interactions compared to water molecule interactions in salt hydrate melts, promoting cellulose dissolution.^12^ E/x/x series: 3.0 wt% CL was dissolved at room temperature under agitation in a 3:7 (v/v) mixture of [EMIm][OAc] and DMSO. Dissolution of cellulose was monitored by light microscopy (NOVEX Holland, B‐Series, 200‐fold magnification).

After reaching a molecularly disperse dissolution of cellulose a vacuum was applied to remove air bubbles from the viscous solutions.

### 2.4 Preparation of Micron‐Porous Cellulose Scaffolds

An overview of the conditions employed in the preparation of nano‐ and dual‐porous cellulose aerogels is given in Table 1.

**Table 1:** Overview of the different combinations of cellulose solvents (Solv_diss_), cellulose concentrations (cotton linters, CL), porogen materials, and solvents used to coagulate cellulose (Solv_coag_) and remove the temporary scaffolds (Solv_leach_)

Sample name	Solv_diss_	CL [wt%]	Porogen	Solv_coag_	Solv_leach_
E/0/x	[EMIm][OAc]/DMSO	3.0	–	EtOH 96%	–
E/W/x	[EMIm][OAc]/DMSO	3.0	Paraffin	EtOH 96%	THF
E/P/x	[EMIm][OAc]/DMSO	3.0	PMMA	EtOH 96%	Acetone
C/0/x	Ca(SCN)_2_ · 8H_2_O/LiCl	1.5	–	EtOH 96%	–
C/P/x	Ca(SCN)_2_ · 8H_2_O/LiCl	1.5	PMMA	EtOH 96%	Acetone

*x* = 0 (no reinforcement), 20, 80 (concentration of the impregnation bath for reinforcement in mg ml^−1^).

Cellulose solutions prepared according to Table 1 were filled into the voids of the temporary porogen scaffolds (paraffin wax or PMMA) which had been prepared beforehand in glass syringes equipped with a removable needle adapter as described earlier. Solutions of cellulose in calcium thiocyanate hydrate undergo sol–gel transition upon cooling below about 80 °C.^13^ Since complete filling of the voids of the fused porogen templates takes about 3 min, whereupon the bottom layers of the cellulose solution are already significantly cooled down due to the long contact pathway at the cold surface of the porogens, the syringes containing the packed beds were preheated to 140 °C to prevent this premature cellulose coagulation and, hence, clogging of voids of the PMMA scaffolds during the filling step. To accelerate cellulose loading, vacuum was applied from the bottom via the tips of the syringes using a commercially available solid phase extraction manifold. After completion of the casting step, the loaded scaffolds were removed from the syringes and immersed in 96% ethanol (sample‐to‐solvent ratio 1/10, v/v) for 24 h to achieve cellulose coagulation. Subsequent simultaneous extraction of the solvent mixture filling the pores of the lyogel and the fused porogen spheres was accomplished by immersion of the scaffold bodies in a large volumetric excess of THF (porogen/paraffin) or acetone (porogen/PMMA) (sample‐to‐solvent ratio 1/20, v/v) and gentle shaking. After five additional solvent replacement/extraction steps (sample‐to‐solvent ratio 1/10, v/v) the samples immersed in THF were transferred either into absolute ethanol (direct scCO_2_ drying) or into acetone (for PMMA reinforcement); in either case three solvent exchange steps were applied.

### 2.5 Reinforcement of Micron‐Porous Cellulose Gels With PMMA

Dual‐porous cellulosic lyogels, whose voids were filled with acetone, were immersed in impregnation baths containing different PMMA concentrations in acetone (20 and 80 mg ml^−1^). After a residence time of at least 24 h, precipitation and drying were simultaneously accomplished by using scCO_2_ as an anti‐solvent and eluent, respectively.

### 2.6 Supercritical Carbon Dioxide Drying

The porogen‐free lyogels were placed into a 300 ml autoclave equipped with a separator for carbon dioxide recycling (SF‐1, Separex, France). Drying was performed under constant flow of scCO_2_ (40 g min^−1^) at 10 MPa and 40 °C for 3 h. The system was then slowly and isothermally depressurized at a rate of <0.1 MPa min^−1^.

### 2.7 Characterization of the Composite Aerogels

Bulk densities have been determined by measuring the dimensions and weight of the nano‐ and dual‐porous aerogels after supercritical carbon dioxide drying.

SEM of gold sputtered samples (Leica EM SCD005 sputter coater, layer thickness 6 nm) was performed on a Tecnai Inspect S50 instrument under high vacuum using an acceleration voltage of 5.00 kV.

Mechanical response profiles towards compressive stress were recorded on a Zwick–Roell Materials Testing Machine Z020. The required strain to achieve a deformation speed of 2.4 mm min^−1^ was measured with a 500 N load cell. Yield strength (*R*_P0.2_) was defined as the stress at 0.2% plastic deformation.

True skeleton densities of the samples were measured by helium gas pycnometry on a Micromeritics Accupyc II 1340 system. Each sample has been measured 400 times at a constant temperature of 27 °C. Based on incertitude of less than 0.3%, a comparison of the two sets of density data allowed determining the void volume fraction of the aerogels.

Thermoporosimetry (TPM) measurements were performed by differential scanning calorimetry (DSC) measurement with a Mettler‐Toledo DSC 823e apparatus equipped with a liquid nitrogen set, using STARe software. The DSC apparatus was calibrated for temperature and enthalpy with metallic standards (In, Pb). The measurement procedure was carried out under air atmosphere using *o*‐xylene as probe solvent.

Nitrogen adsorption/desorption experiments have been conducted at 77 K on a Quantachrome Autosorb1 device. All samples were degassed in vacuum prior to analysis. Specific surface areas were calculated using the Brunauer, Emmett, and Teller (BET) equation with 11 points.

#### 2.7.1 Biocompatibility Testing

Dual‐porous aerogel discs (10 mm diameter, 2 mm height) were sterilized with UV light for 30 min from both plane sides. All samples used for biocompatibility testing were prepared using the porogen size fraction 200–300 μm.

Cell seeding and Cultivation: NIH 3T3 fibroblast cells were seeded in 100 μl cell suspension containing 6000 cells per sample. To allow cell attachment, the plates were incubated for 1 h (37 °C, 5% CO_2_) before adding 900 μl cell culture medium (DMEM containing 10% NCS and 1% P/S).

*MTT staining and assay*: Cell viability in the dual‐porous cellulose scaffolds was determined after 3 and 7 d of cultivation time by the MTT assay (*n* = 6). The culture medium was withdrawn from the samples and the cells were rinsed with PBS. 1 ml of working solution (10% MTT and 90% basal medium) were added to every sample in a 24‐well plate and incubated for 4 h at 37 °C. To dissolve the formazan crystals formed by the cells 900 μl SDS in 0.01 M HCl was added. The plate was then further incubated for at least 24 h at 37 °C. Subsequently, the absorbance of the solution was measured by a microplate photometer (Thermo scientific Multiskan FC) at 570 nm (absorbance maximum) and at 630 nm (to determine the effect of the medium). Statistical analysis was performed using a Student's *t*‐test (two‐tailed, p < 0.05).

*DAPI staining*: To evaluate the distribution of cells across the surface of the scaffolds, the double‐stranded DNA (dsDNA) of the cells nuclei was stained with the fluorescent marker DAPI. After 3 and 7 d of cultivation, the cells were first rinsed with PBS and then fixed by incubation in ice‐cold 96% EtOH. After rinsing with PBS again, the samples were covered in DAPI solution and incubated for 15 min at 37 °C in the dark. The samples were then rinsed and covered with PBS. Microscopic analysis (Leica DM IL LED) was carried out at the maximum fluorescence emission of the DAPI/dsDNA complex (460 nm).

*SEM analysis of cell cultures*: For selected samples (E/P/0, C/P/0, C/P/20): After the cells were fixed with ice‐cold 96% EtOH as described for DAPI staining, a gradual solvent exchange from PBS to absolute ethanol was carried out (increment of 10 vol%, 6 h per step). Absolute ethanol was exchanged two more times and the alcogels were then dried by scCO_2_ extraction. The cell cultures found on the dual‐porous aerogel scaffolds after 7 d of cultivation were analyzed by SEM under the conditions described above.

## 3 Results and Discussion

Scaffolds composed of microfibrillary nanoporous cellulose struts embedding interconnected micron‐size pores were obtained by consecutive vacuum casting of respective cellulose solutions into the voids of a temporary scaffold consisting of fused paraffin or PMMA micronspheres, cellulose coagulation, exhaustive porogen leaching, as well as scCO_2_ drying of the resulting cellulose lyogels.

**Figure 2:**
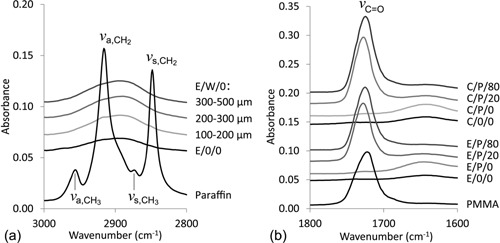
ATR‐IR spectra of nanoporous versus dual‐porous cellulose aerogels after leaching of the temporary paraffin wax ((a); no reinforcement, different porogen size fractions) and PMMA templates ((b); different cellulose solvents (E, C), porogen size fraction 100–200 μm, non‐reinforced and reinforced samples, numbers in the sample label represent the PMMA concentration of the impregnation bath in mg ml^−1^).

Leaching of the temporary porogen scaffold was followed by ATR‐IR spectroscopy, measured at a central position of the aerogels cylinders (Figure 2). A comparison of E/W/0 samples (E, cellulose solvent [EMIm][OAc]; W, paraffin wax porogens; 0, no reinforcement with PMMA) of different micron‐size porosity (100–200, 200–300, 300–500 μm) confirmed quantitative removal of the temporary scaffold as all signals caused by paraffin—CH_2_ and CH_3_ stretching vibrations at 2848, 2916 and 2874, 2956 cm^−1^, respectively—had disappeared after leaching the wax matrix. Likewise, the spectral range of ester‐type carbonyl stretching vibrations (1725 cm^−1^) confirmed the complete removal of the porogen material from the samples E/P/0 and C/P/0 (C, cellulose solvent Ca(SCN)_2_/H_2_O/LiCl; P, PMMA porogens; 0, no reinforcement with PMMA), which were prepared using the respective PMMA‐based temporary scaffolds instead of the paraffin wax counterparts (Figure 2b).

FT‐IR furthermore confirmed successful reinforcement of the micronporous aerogels with PMMA for both of the studied solvent systems (E/P/20 and C/P/20; E/P/80 and C/P/80; numbers represent the PMMA concentration of the impregnation bath in mg ml^−1^). This is in good agreement with our previous study, investigating the reinforcement of bacterial cellulose (BC) aerogels with biocompatible polymers, such as poly(lactic acid) (PLA), polycaprolactone (PCL), PMMA, and cellulose acetate.^14^ Impregnation of respective BC lyogels with PMMA solutions in acetone and subsequent scCO_2_ anti‐solvent precipitation of the secondary polymer had been demonstrated to be a suitable approach to create an interpenetrating, homogeneous network of reinforcing PMMA. The existence of the latter had been confirmed by cellulose extraction with [EMIm][OAc] and subsequent scanning electron microscopic investigations, revealing a PMMA morphology resembling that of the original BC aerogel.

The generation of micron‐size porosity significantly reduces the bulk densities (*ρ*_B_) of non‐reinforced cellulosic aerogels. While common, micronpore‐deficient cellulose II aerogels obtained by ethanol‐mediated cellulose coagulation from solution state and subsequent scCO_2_ drying, had densities of 34.1 mg cm^−3^ (1.5 wt% cotton linters (CL) in Ca(SCN)_2_ · 8H_2_O:LiCl = 97.5:2.5, w/w) and 55.8 mg cm^−3^ (3.0 wt% CL in [EMIm][OAc]:DMSO = 3:7, vol/vol), their dual‐porous counterparts were significantly less dense reaching values of down to 14 mg cm^−3^ (Table 2). Reinforcement with PMMA inevitably re‐increases the materials' density. The gain in bulk density correlates with the size of the used porogen spheres, as demonstrated for the fractions 100–200 and 200–300 μm. For both of the tested degrees of PMMA reinforcement (20, 80, cf. above), higher bulk densities were obtained for aerogels prepared from temporary scaffolds which were composed of fused porogen spheres 200–300 μm in diameter, independent of the type of cellulose solvent (E, C) and porogen material (W, P).

**Table 2:** Bulk densities (*ρ*_B_) of cellulose aerogels differing in cellulose solvent (E, C), porogen material (W, P), micronporosity, and degree of reinforcement by an interpenetrating PMMA network

		ρ_B_ [mg cm^−3^]; *n* = 5 PMMA in the impregnation bath in mg ml^−1^		
Sample name	Porogen size fraction [μm]	*x* = 0	*x* = 20	*x* = 80
E/0/x	porogen‐free	55.8 ± 0.4		
E/W/x	100–200	23.1 ± 2.6^[a]^	34.6 ± 7.9^[b]^	99.7 ± 9.5^[b]^
E/W/x	200–300	18.8 ± 1.0	47.7 ± 5.9^[c]^	132.7 ± 10.2^[c]^
E/W/x	300–500	20.2 ± 3.5^[c]^		
E/P/x	100–200	17.8 ± 1.4	35.0 ± 1.7	107.5 ± 1.4
E/P/x	200–300	19.1 ± 2.0^[c]^	44.7 ± 6.8^[c]^	187.7 ± 5.3^[c]^
C/0/x	porogen‐free	34.1 ± 0.8^[d]^		
C/P/x	100–200	14.4 ± 3.3	28.0 ± 2.7	102.8 ± 8.2
C/P/x	200–300	15.3 ± 0.9^[c]^	31.0 ± 4.6^[c]^	207.6 ± 9.0^[c]^

a *n* = 9.

b *n* = 6.

c *n* = 4.

d *n* = 7.

Selected micronporous cellulose aerogels (porogen size fraction 100–200 μm), differing in cellulose solvent/porogen combinations, were tested with regard to a possible *ρ*_B_ gradient in longitudinal direction of the cylindrical specimen. This could have been caused by either inhomogeneous distribution of forces over the length of the porogen molds (∼2.5 cm) during unidirectional compression of the porogen spheres, or through gradual alteration of the templating matrix during loading of the cellulose solution. The latter was particularly likely to happen for all C/P samples, as loading of the temporary PMMA scaffold with solutions of cellulose in Ca(SCN)_2_ · 8H_2_O is accomplished at 140 °C which is considerably above the glass transition temperature of the used PMMA spheres (*T*_g_ = 105 °C).

Upper and lower sections (according to their position during the porogen compression and filling procedure) of E/W‐ and E/P‐derived micronporous aerogels showed a negligible difference in density (density of lower sections ∼1% higher). This confirms that the compressive forces occurring during preparation of the packed beds of paraffin wax and PMMA spheres were well distributed in direction of the applied stress. The somewhat lower density (about 8%) at the bottom of the C/P samples is therefore most likely due to the comparatively high temperature of the cellulose solution during vacuum‐assisted filling of the PMMA templates, which is necessary to prevent premature cellulose coagulation in calcium thiocyanate octahydrate, but seems to soften PMMA to some extent. At the bottom and hence on the vacuum side of the PMMA template, shear forces thus caused a slight compaction of the template, leading to a somewhat reduced volume filled with cellulose solution and hence a lower density of the resulting aerogel.

### 3.1 Morphology

All tested combinations of process variables, such as 1) solvent system used for cellulose dissolution, 2) type and size of porogen spheres, 3) type of solvent used to extract the porogen after cellulose coagulation, and 4) degree of reinforcement, yielded aerogels of the desired dual network morphology, that is, a nanoporous, partly PMMA‐reinforced 3D network of cellulose struts embedding interconnected micron‐size pores.

**Figure 3:**
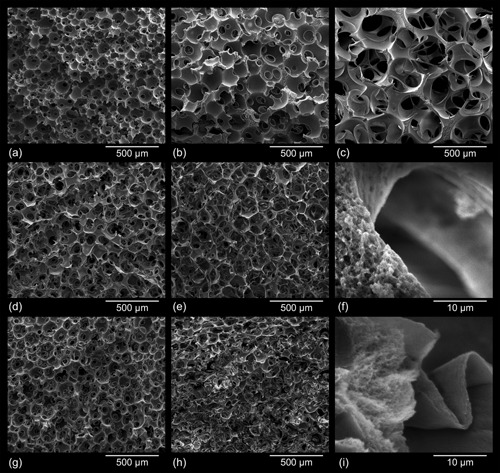
SEM micrographs of dual‐porous cellulosic aerogels prepared using different cellulose solvent/porogen combinations: 3.0 wt% CL in [EMIm][OAc]/DMSO (3:7, vol/vol), template of fused paraffin spheres (E/W)—porogen size 100–200 μm (a), 200–300 μm (b), and 300–500 μm (c); 3.0 wt% CL in [EMIm][OAc]/DMSO, template of fused PMMA spheres (E/P)—porogen size 100–200 μm (d) and 200–300 μm (e); 1.5 wt% CL in Ca(SCN)_2 _· 8H_2_O/LiCl (97.5/2.5, w/w), scaffold of fused PMMA spheres (C/P)—porogen size 100–200 μm (g) and 200–300 μm (h); scaffold strut morphologies of E/W/0 (f) and C/P/0 (i) aerogels.

However, SEM pictures revealed that the morphological features of the cellulose network initially formed within the voids of the temporary porogen scaffold are preserved to a different extent during subsequent leaching of the temporary scaffold and scCO_2_ drying, depending on the type of porogen used. While a far‐reaching preservation of the dual porous cellulose network was possible for the temporary paraffin scaffold (E/W aerogels, see Figure 3a–c), the morphology of the cellulose scaffold struts was substantially altered during leaching of the fused PMMA spheres. Macroscopic fractures present along the sample bodies of E/P and C/P are indicative of solvent‐induced cracking occurring during leaching of PMMA with acetone.^15^ This is assumed to be due to an expansion of PMMA that can reach 19% in saturated acetone vapor.^16^ Compression of the fragile cellulose struts furthermore modifies their nanoporosity and expands the interconnections between individual micron‐size pores.

As discussed earlier, slight deformation of the temporary PMMA scaffold caused by the high temperature required for cellulose dissolution in Ca(SCN)_2_ · 8H_2_O caused a density gradient with the lowest *ρ*_B_ values on the bottom of the samples. Accordingly, morphological defects in terms of a somewhat deformed scaffold structure were observed by SEM of some C/P samples (e.g., Figure 3h).

**Figure 4:**
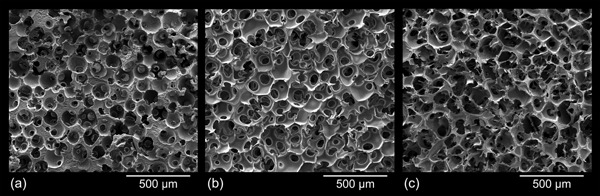
SEM micrographs of dual‐porous cellulose aerogels (porogen size 100–200 μm) reinforced by impregnation with PMMA at the lyogels stage using impregnation bath concentrations of 20 mg ml^−1^ (a: E/W/20; b: E/P/20) and 80 mg ml^−1^ of PMMA in acetone (c: E/P/80).

SEM analysis of breaking edges of the scaffolds struts furthermore confirmed that the cellulose struts themselves consist of a secondary network of smaller interconnected voids comprising the full nano and low micron range (Figure 3f and i). The morphology of these cellulose II networks is largely affected by both the type of cellulose solvent and the type of anti‐solvent used to coagulate cellulose from solution state. Cellulose coagulation from solutions in Ca(SCN)_2_/H_2_O/LiCl using ethanol as anti‐solvent yielded solely fibrillary networks while their counterparts derived from solutions in [EMIm][OAc]/DMSO featured an additional globular superstructure. This observation is in good agreement with the morphologies of respective nanoporous aerogels^17^ and a recent study that proposed different phase separation mechanisms for cellulose coagulated from aqueous 8 wt% NaOH or solidified NMMO · H_2_O (two‐stage mechanism) and from ionic liquids or molten NMMO · H_2_O (spinodal decomposition in one step).^18^ Reinforcement of the dual‐porous cellulose aerogels—accomplished by immersing the lyogels in acetone solutions of low (20 mg ml^−1^) or high (80 mg ml^−1^) PMMA concentration after porogen leaching and subsequent scCO_2_ drying—originated materials of considerably improved mechanical stability (cf. Mechanical properties). The morphology of the initially formed pure cellulose network is largely maintained only at the lower PMMA impregnation level (Figure 4a and b), with PMMA solely precipitating on the surfaces of the cellulose fibrils. At the higher level PMMA forms a secondary interpenetrating network which is partially blocking the interconnections between the micronpores. Besides that, the spherical shape of the micronpores is somewhat impaired due to precipitation of PMMA within the voids. However, the composites are still largely open‐porous and their mechanical properties are greatly improved compared to those of low PMMA content.

### 3.2 Mechanical Properties

Nanoporous cellulose aerogels are lightweight, ductile materials. The introduction of additional large, interconnected micron‐sized pores was therefore expected to have a particularly negative impact on the mechanical properties, such as compressive modulus and strength, as observed for other materials.^2^, ^19^ This has been confirmed in the current study: the Young's moduli (*E*_compr_) of non‐reinforced micronporous E/W/0 aerogels decreased by about 70–80% compared to their respective counterparts without micron‐size pores, depending on the pore size. Similarly, yield strength dropped by more than one order of magnitude, reaching values of about 10 kPa only (Table 3).

**Table 3:** Mechanical properties of dual‐porous cellulose‐based aerogels and composites under compressive stress (*n* = 4): Young's modulus (*E*), yield strength (*σ*) and specific modulus (*E_ρ_*). The last digit of the sample name indicates the PMMA concentration in the impregnation bath in mg ml^−1^

Sample name	Porogen size fraction [μm]	*E*_compr_ [MPa]	*σ* [kPa]	*E_ρ_* [MPa cm^3^ g^−1^]
E/0/0	porogen‐free	1.12 ± 0.21^[a]^	114.8 ± 11.2^[a]^	20.1 ± 3.7^[a]^
E/W/0	100–200	0.31 ± 0.13^[a]^	12.4 ± 1.8^[a]^	13.6 ± 5.4^[a]^
E/W/0	200–300	0.33 ± 0.12	11.7 ± 3.3	17.7 ± 6.3
E/W/0	300–500	0.20 ± 0.10^[a]^	7.1 ± 1.4^[a]^	8.8 ± 3.0^[a]^
E/W/20	100–200	1.11 ± 0.26	62.7 ± 20.8	32.1 ± 7.5
E/W/80	100–200	8.76 ± 0.91	337.2 ± 52.5	87.9 ± 9.2
E/P/0	100–200	0.24 ± 0.06^[a]^	16.5 ± 6.3^[a]^	13.3 ± 3.1^[a]^
E/P/20	100–200	1.02 ± 0.21	49.9 ± 19.6	29.2 ± 6.1
E/P/80	100–200	8.32 ± 1.56	320.7 ± 26.9	77.4 ± 14.5
C/0/0	porogen‐free	2.00 ± 0.07^[a]^	76.4 ± 2.4^[a]^	66.6 ± 2.1^[a]^
C/P/0	100–200	0.10 ± 0.03	7.7 ± 4.2	6.8 ± 4.0
C/P/20	100–200	0.66 ± 0.11	39.3 ± 10.7	23.7 ± 9.8
C/P/80	100–200	5.59 ± 1.01	216.7 ± 40.0	54.3 ± 3.1

a *n* = 5.

Even though there are various opportunities to improve the mechanical properties of lightweight, fragile cellulose aerogels under preservation of their “all‐cellulose” character, such as using different cellulose allomorphs (Iα, Iβ, II), degree of crystallinity, fibrillation, and polymerization, micronporous cellulose aerogels of ultralow density require reinforcing strategies capable of improving the mechanical properties in a more pronounced way.^17^ Impregnation of bacterial cellulose aerogels with a solution of PMMA in acetone and subsequent coagulation of the biocompatible polymer using scCO_2_ as an anti‐solvent has previously been confirmed to be a suitable approach to reinforce the largely nanoporous BC fibril network under preservation of its open‐porous morphology.^14^ In this work, the same method has been tested as a means to reinforce the obtained dual‐porous cellulose gels with interpenetrating PMMA networks. A comparison of the mechanical properties (compression tests) of dual‐porous aerogels prior to and after reinforcement revealed that already at the lowest PMMA concentration in the impregnation bath (20 mg ml^−1^) both stiffness (Young's modulus *E*) and strength (*σ*) increased by factor 4–5 for most of the tested samples. Strong reinforcement using a PMMA impregnation bath concentration of 80 mg ml^−1^ generates materials having *E* moduli of 5.6 MPa (C/P/80) to 8.8 MPa (E/W/80), exceeding that of the respective PMMA‐free samples by a factor of 30–60. Density‐normalized Young's modulus (*E* *_ρ_*)—a convenient parameter to compare the stiffness of materials of varying density – increased by factor 2.2–3.5 for the low and by factor 6.5–8.0 for the high level of reinforcement (Table 3). In case of the dual‐porous aerogels obtained by coagulation of cellulose from respective solutions in [EMIm][OAc] at room temperature (E/W and E/P samples), the specific modulus of the micronpore‐deficient reference materials (E/0/0) was exceeded already using a PMMA impregnation bath concentration of 20 mg ml^−1^.

Common micronpore‐deficient aerogels prepared from solutions of 1.5 wt% cotton linters in a Ca(SCN)_2_ · 8H_2_O salt hydrate melt which contained 2.5 wt% of LiCl were confirmed to be significantly stiffer (*E_ρ_* = 66.6 ± 2.1 MPa cm^3^ g^−1^) than samples obtained from twice the amount of cellulose dissolved in the room temperature ionic liquid [EMIm][OAc] (*E_ρ_* = 20.1 ± 3.7 MPa cm^3^ g^−1^). This explains the surprisingly high dimensional stability of lyogels prepared by coagulation of cellulose from solutions in Ca(SCN)_2_ during their conversion to aerogels, which has already been reported in the early days of cellulose aerogel research.^12^ Different from other approaches which use cellulose solvents such as NMMO · H_2_O, aqueous NaOH, ionic liquids, or DMSO/TBAF, associated with strong shrinkage easily exceeding 30 vol% for gels from 3 wt% cellulose solutions, Ca(SCN)_2_‐derived gels suffer only little shrinkage. All the more it was surprising that the creation of micron‐size pores (100–200 μm) using PMMA spheres caused a much more severe drop in *E_ρ_* modulus for the C/0/0 and C/P/0 sample pair (66.6–6.8 MPa cm^3^ g^−1^) than for the actually more fragile counterparts obtained from respective cellulose solutions in [EMIm][OAc] (20.1–13.3 MPa cm^3^ g^−1^). Reinforcement of the C/P/0 samples was not able to regain the density‐normalized *E* module of the micronpore‐deficient C/0/0 aerogel even at the highest tested PMMA concentration of the impregnation bath, even though the Young's modulus of C/P/80 was somewhat higher (5.59 MPa) compared to the C/0/0 sample (2.00 MPa). It is assumed that structural inhomogeneity, which was induced during filling of the PMMA porogen mold with the hot solution of cellulose in Ca(SCN)_2_ (also cf. Morphology), affects the aerogels mechanical properties.

### 3.3 Porosity and Internal Surface Area

Helium gas pycnometry confirmed that complementing of nanoporous cellulose II aerogels with interconnected micron‐size pores causes a slightly increased total porosity compared to the reference materials, independent of the type of cellulose solvent or porogen used. This is evident from a comparison of respective sample pairs deficient/rich in micron‐size pores, such as E/0/0 versus E/W/0 ([EMIm][OAc], wax spheres), E/0/0 versus E/P/0 ([EMIm][OAc], PMMA spheres), and C/0/0 versus C/P/0 (Ca(SCN)_2_, PMMA spheres), see Table 4. Pore volume fractions of >98% for micronporous cellulose aerogels are in agreement with a study in which PMMA particles (porogen size fractions between 120 and 500 μm) were mixed into 1.72 wt% solutions of softwood pulp in 1‐butyl‐3‐methylimidazolium chloride and *N*,*N*‐dimethylacetamide/LiCl, respectively. After cellulose coagulation and extraction of the solvent, the cellulose/porogen mixtures were oven‐dried (40 °C) and PMMA was thereafter leached by dichloromethane.^6^ The impact of the porogen size on the specific surface area of the resulting aerogels has been exemplarily studied for the E/W/0 series, as removal of the temporary paraffin scaffold does not alter the internal lyogel structure of the scaffold struts. Compared to the micronpore‐deficient reference aerogels, the specific surface area (SSA) values of the dual‐porous aerogels decreased with increasing size of the porogens by 1.2% (100–200 μm), 32.5% (200–300 μm), and 44.7% (300–500 μm). This effect is assumed to be due to a variation of the cellulose II morphology at the strut/micronpore interface, compared to the internal strut morphology. A denser network was formed in regions where cellulose was in close contact to the porogen surface during regeneration (cf. Figure 3f). This could be the result of repulsive forces between the rather polar surface hydroxyl groups of cellulose and the non‐polar surface of the porogen materials, as previously observed for Teflon or rubber molds used during cellulose regeneration.^20^ When PMMA was used as a porogen (E/P/0), the SSA already decreased by 59.3% at the size fraction 100–200 μm. This reduction was caused by a compaction of the cellulose fibril network, which occurred due to swelling of PMMA during the porogen leaching process, and is also evident from the respective scanning electron micrographs (cf. Morphology, Figure 3i).

**Table 4:** Porosity, specific surface area (SSA) and surface area‐to‐volume ratio (SA*_V_*) of common nanoporous aerogels in comparison to dual nano‐/micronporous aerogels (porogen size 100–200 μm) prior to and after reinforcement with PMMA

Sample name	Porogen size fraction [μm]	Porosity [%]	SSA [m^2^ g^−1^]	SA*_V_* [m^2^ cm^−3^]
E/0/0	porogen‐free	96.3	246 ± 2	13.7 ± 0.1
E/W/0	100–200	98.5	243 ± 8	5.6 ± 0.2
E/W/0	200–300	98.8	166 ± 5	3.1 ± 0.1
E/W/0	300–500	98.7	136 ± 3	2.8 ± 0.1
E/W/20	100–200	97.5	188 ± 2	6.5 ± 0.1
E/W/80	100–200	92.1	56 ± 1	5.6 ± 0.1
E/P/0	100–200	98.8	100 ± 2	1.8 ± 0.0
E/P/20	100–200	97.4	115 ± 4	4.0 ± 0.1
E/P/80	100–200	91.4	53 ± 2	5.7 ± 0.2
C/0/0	porogen‐free	97.8	190 ± 4	6.5 ± 0.1
C/P/0	100–200	99.1	58 ± 5	0.8 ± 0.1
C/P/20	100–200	98.5	77 ± 5	2.2 ± 0.1
C/P/80	100–200	91.7	20 ± 1	2.1 ± 0.1

Reinforcement of the obtained dual‐porous aerogels by interpenetrating PMMA networks consistently produced materials of reduced porosity, an effect that increased with the degree of reinforcement and was independent of the type of cellulose solvent or porogen used. Aerogels obtained from a porogen size fraction of 100–200 μm suffered a porosity reduction of 0.6–1.4% at the lowest (20 mg ml^−1^) and 6.4–7.4% at the highest reinforcement level (80 mg ml^−1^; Table 4).

The interconnected nanoporosity of the cellulose struts forming the micronporous scaffolds and hence the achievement of dual porosity for all aerogels was confirmed by thermoporosimetry (TPM), which has been previously demonstrated to be a powerful analytical tool to reveal the pore characteristics of highly fragile lightweight cellulosic aerogels.^14^ TPM allows the determination of the pore size distribution (PSD) of open porous materials in the full nanometer scale up to about 20 μm. TPM investigates the shift of either the melting (*T*_m_) or crystallization temperature (*T*_c_) of a suitable interstitial liquid, caused by its confinement in small pores compared to the respective *T*_m_ or *T*_c_ of the “free” solvent. Both the extent of freezing point depression and shape of the heat flow curve obtained by differential scanning calorimetry largely depend on the pore size and the PSD, respectively.^21^

**Figure 5:**
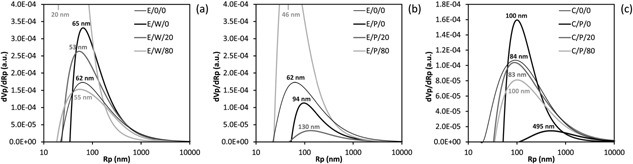
Thermoporosimetry analysis: Pore size distribution (<10 μm) of nanoporous and dual‐porous aerogels of the E/W (a), E/P (b), and C/P (c) series with and without PMMA reinforcement.

While the obtained PSDs (Figure 5) were affected by both, introduction of micron‐size pores and PMMA reinforcement, they generally covered a broad range of meso‐ and macropores with radii ranging from about 10 nm to 10 μm with maxima at 20–130 nm. For sample C/P/0 an additional peak was observed at about 500 nm which is indicative for the density gradient generated during loading/coagulation of cellulose inside the packed beds of PMMA spheres as discussed earlier. TPM furthermore confirms the impact of PMMA swelling on the morphology of the scaffolds, an effect that happens during leaching of the PMMA spheres with acetone. While all cellulose solvent/porogen material combinations resulted in a loss of the smallest pore size fractions compared to the reference samples free of micron‐size pores, in case of paraffin wax the peak position shifted only negligibly compared to the reference aerogels (62 → 65 nm). In contrast, swelling of PMMA templates caused a considerable shift towards larger pore sizes (E/P: 62 → 94 nm, C/P: 84 → 100 nm) as a result of the strut compaction. The final PMMA reinforcement step preserved dual‐porous morphologies for all studied reinforcement levels.

Nitrogen sorption experiments at 77 K furthermore confirmed that the introduction of a secondary PMMA network, which penetrates the primary nanoporous cellulose strut network embedding micron‐size pores, yields materials of lower SSA compared to non‐reinforced samples (Table 4). This is in good agreement with our previous study that investigated the reinforcement of bacterial cellulose aerogels with biocompatible polymers, such as PCL, PLA, cellulose acetate, and PMMA.^14^ The same study proposed normalization of internal surface area for sample volume (SA*_V_*) rather than for mass (SSA) as a more meaningful approach to evaluate the effect of a reinforcing step on the accessible surface area, as the introduction of a secondary polymer inevitably leads to a gain in weight. In the current study, it has been shown that SA*_V_* decreased for all samples upon generation of micron porosity. However, if the micronporous lyogels are reinforced with PMMA prior to scCO_2_ drying, the drop in SA*_V_* values is significantly less pronounced. This confirms the results of compression tests that clearly revealed improved mechanical stability for the PMMA‐reinforced dual‐porous aerogels and, hence, better preservation of the hierarchical architecture of the cellulosic network formed by coagulation from solution state.

### 3.4 Biocompatibility

**Figure 6:**
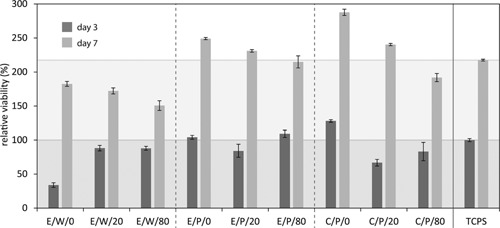
MTT assay: Cell growth and proliferation on dual‐porous aerogels after 3 and 7 d of cultivation (*n* = 6) as evaluated by MTT assay and compared with clinically used tissue polystyrene (3 d = 100%).

All aerogels obtained in the current study can be considered as promising cell scaffolding materials for various tissue engineering applications due to their multi‐scale, interconnected porosity, microtopology of the nanoporous cellulose struts, and particular mechanical characteristics. To pre‐evaluate their biocompatibility, the viability of fibroblast cells seeded on the surface of selected dual‐porous aerogel samples (porogen size 200–300 μm) was investigated with a MTT assay after 3 and 7 d of *in vitro* cultivation, respectively. For most samples robust cell growth was observed already after 3 d of cultivation (Figure 6). In particular, the micron‐porous aerogels prepared using packed beds of fused PMMA spheres as temporary templates exhibited excellent biocompatibility. Here, the viability of cells on samples of the series E/P/0, E/P/20, C/P/0, and C/P/20 was significantly better after an incubation period of 7 d compared to the clinically used tissue culture polystyrene (TCPS, 100% at day 3), which was included in this study as a reference material. Even though MTT activity on the dual‐porous cellulose aerogels decreases with the extent of PMMA reinforcement, cell viability of the mechanically sufficiently stable E/P/20 (231%) and C/P/20 (240%) variants was still significantly higher than that of the reference material at day 7 (218%). Compared to micron‐porous samples prepared using temporary PMMA templates, their counterparts obtained from paraffin wax molds showed distinctly lower cell viability, not exceeding ca. 85% of TCPS at the end of the cultivation period. As both types of porogen scaffolds are quantitatively removed during the leaching process (Figure 2) and identical cellulose solvent/anti‐solvent combinations were used in case of the E/x/x series, the different morphologies entailed by the porogen materials remain the only plausible reason for the different cell viabilities observed for these two types of aerogels (E/W/x vs. E/P/x). The somewhat lower cell viability on aerogels derived from cellulose solutions in [EMIm][OAc] (E/P/0) compared to those in calcium thiocyanate (C/P/0) could be due to covalent immobilization of 1‐ethyl‐3‐methyl‐imidazolium moieties at the reducing ends of cellulose (cf. 22), and the well‐known ecotoxicity of many 1*H*‐imidazolium derivatives. Other factors that may have an impact as well are aerogel density, surface topology, and degree of cellulose crystallinity.

The introduction of PMMA decreases MTT activity after 7 d of cell culture. Interestingly, samples E/P/20 and C/P/20 show a stronger increase in viability between days 3 and 7 compared to their non‐reinforced counterparts.

**Figure 7:**
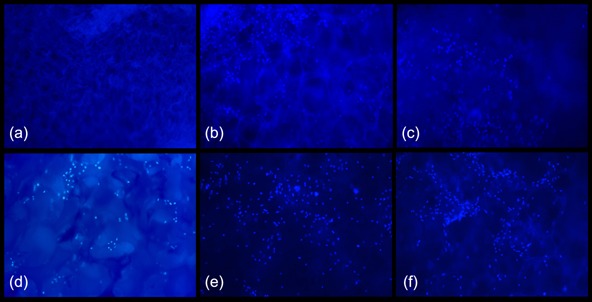
Fluorescence (*λ*_ex_ = 460 nm, 100‐fold magnification) micrographs of fibroblast cells on the surface of dual‐porous cellulose aerogels after 7 d of cultivation and visualization by DAPI staining. E/W/0 (a), E/P/0 (b), C/P/0 (c), E/W/80 (d), E/P/80 (e), and C/P/20 (f).

Visualization of NIH 3T3 fibroblast cells after 7 d of cultivation on the dual‐porous cellulosic scaffolds—accomplished by 4′,6‐diamidino‐2‐phenylindole (DAPI) staining of their cell nuclei and subsequent fluorescence microscopy—revealed that E/P/0, E/P/80, C/P/0, and C/P/20 were the most promising samples with regard to cell attachment and proliferation (Figure 7). For samples in which paraffin was used as a porogen, only moderate cell attachment was observed after PMMA reinforcement (cf. samples E/W/0 vs. E/W/80; Figure 7a and d). This suggests that the alteration of the scaffold strut morphology by porogen swelling during the leaching step as observed for the E/P/0 and C/P/0 samples may have a positive impact on cell adhesion.

**Figure 8:**
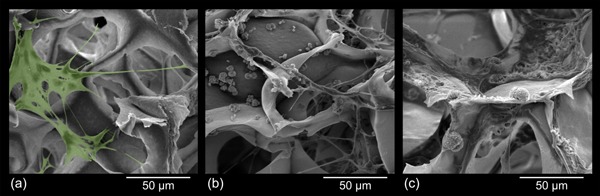
SEM micrographs of dual‐porous cellulosic aerogels seeded with NIH 3T3 fibroblast cells confirmed good cell attachment and spreading after 7 d of cultivation. E/P/0 (a), C/P/0 (b), and C/P/20 (c). The green region in (a) (colorized using Adobe Photoshop) illustrates a cell spreading across micron‐size pores and adhering to the nano‐porous struts of the cellulosic aerogel scaffold.

The shape of cells is an important factor that decides about whether cells live and proliferate or suffer apoptosis (programmed cell death). Depending on their micro‐environment, which includes local cell density and ECM compatibility in terms of chemical, morphological, and mechanical properties, cells either spread and flatten—a prerequisite to cell survival and growth—or maintain a spherical shape and enter apoptosis.^23^ SEM images of NIH 3T3 fibroblast cells, cultivated on selected dual‐porous aerogels show a considerable amount of cells spreading either across the micron‐size pores (Figure 8a and b, samples E/P/0, C/P/0) or adhering on the surface of the nano‐porous cellulose struts (Figure 8c, sample C/P/20). The highest number of cells was found on the non‐reinforced scaffolds C/P/0 (Figure 8b) prepared from cellulose solutions in calcium thiocyanate octahydrate supplemented with 2.5 wt% of LiCl.

However, the combination of cellulose with a small amount of PMMA seems to promote cell attachment and spreading, which explains the slightly increased proliferation observed for samples C/P/20 and E/P/20 between days 3 and 7 of cultivation (cf. Figure 6). This enhanced cell response is assumed to be due to the generation of medium hydrophobic PMMA surfaces. While neither extreme is beneficial, a series of studies have confirmed enhanced protein adsorption and cell attachment for ECM materials exhibiting both hydrophilic and hydrophobic surface properties.^24^ The nevertheless good cell response observed for the entirely cellulose‐based scaffolds E/P/0 and C/P/0 is assumed to be due to the presence of less polar regions in the cellulose II polymorph caused by effects similar to the sheet‐like assembly of cellophane observed in the presence of hydrophobic cellulose anti‐solvents.^25^ Yet, as a consequence of the high abundance of hydroxyl groups, the hydrophilic character dominates the overall amphiphilicity of the material.^26^ By introducing hydrophobic PMMA as a secondary polymer, scaffolds of mixed polarity are obtained, resulting in improved cell attachment and spreading.

## 4 Conclusion

Cellulose‐based aerogels of dual‐porosity have been prepared by coagulating cellulose solutions within the voids of a temporary template of fused porogen spheres, subsequent leaching of the porogens and supercritical carbon dioxide drying of the obtained lyogels containing interconnected micron‐size pores embedded within networks of nanoporous cellulose struts. Variations in porogen characteristics, type of cellulose solvent and extent of impregnation with a secondary biocompatible polymer (PMMA) have been investigated as tools to tailor cellulosic aerogels for cell scaffolding applications.

Both the cellulose solvent and anti‐solvent used to dissolve and coagulate cellulose from solution state have previously been confirmed to be key factors governing the morphology and nano‐scale void characteristics of cellulose II aerogels. While the cellulose solvent Ca(SCN)_2_/H_2_O/LiCl in combination with the anti‐solvent ethanol yields solely fibrillary cellulose networks, an additional globular superstructure is formed when the room temperature ionic liquid [EMIm][OAc]/DMSO is used for cellulose dissolution.

Interconnected micron‐size porosity can be added to regular nanoporous cellulose II aerogels using packed beds of fused micron‐size paraffin wax or PMMA porogen spheres. After filling the voids of these temporary templates with cellulose solution, an anti‐solvent is added and the porogen is leached using an appropriate organic solvent. Preservation of the specific dual‐porous cellulose II morphology throughout the subsequent process steps, namely porogen leaching, solvent exchange, and scCO_2_ drying, largely depends on type and size of the porogen spheres, as well as their compatibility with the conditions required to achieve cellulose dissolution. In this respect, the combination of temporary scaffolds from fused micron‐size paraffin wax spheres with cellulose solutions in room temperature ionic liquids turned out to produce better aerogels in terms of homogeneity than the combination of PMMA templates with cellulose solutions in Ca(SCN)_2_/H_2_O/LiCl. This is mainly due to softening of PMMA at the high temperature (140 °C) required during the cellulose loading step, which results in longitudinal density gradients and slight morphological defects within the aerogels. Measures to overcome this drawback are subject of ongoing research, including approaches to reduce the temperature of cellulose dissolution without causing a strong increase in viscosity or using porogen materials of higher glass transition temperature.

The type of porogen also affects the extent to which the cellulose network morphology can be preserved during removal of the temporary template. While the morphology is well preserved for paraffin wax spheres, swelling of PMMA during leaching causes compaction of the nanoporous cellulose struts, and thus widening of the interconnections between the micron‐size pores.

The loss in compressive modulus and yield strength due to the introduction of micron‐size porosity can be easily compensated at full preservation or even increase of the surface area‐to‐volume ratio by reinforcing the lyogels with a secondary network of PMMA employing scCO_2_ anti‐solvent techniques. Following this approach, the density‐normalized modulus could be increased by a factor of 2.2–3.5 for the lower and by a factor of 6.5–8.0 for the higher PMMA concentration of the impregnation bath.

MTT biocompatibility tests investigating viability and proliferation of fibroblasts cells seeded on the surface of the obtained dual‐porous aerogels, the materials prepared from calcium thiocyanate octahydrate/LiCl solutions, and temporary PMMA porogen scaffolds had the best biocompatibility amongst the tested parameter combinations in terms of cell viability, distribution, and spreading.
